# Endodontic and Esthetic Management of a Dilacerated Maxillary Central Incisor Having Two Root Canals Using Cone Beam Computed Tomography as a Diagnostic Aid

**DOI:** 10.1155/2014/861942

**Published:** 2014-05-18

**Authors:** Sarang Sharma, Shibani Grover, Vivek Sharma, Dhirendra Srivastava, Meenu Mittal

**Affiliations:** ^1^Department of Conservative Dentistry and Endodontics, ESIC Dental College and Hospital, Sector 15, Rohini, Delhi 110089, India; ^2^Department of Oral Surgery, ESIC Dental College and Hospital, Sector 15, Rohini, Delhi 110089, India; ^3^Department of Pediatric Dentistry, ESIC Dental College and Hospital, Sector 15, Rohini, Delhi 110089, India

## Abstract

Traumatic injuries to the primary dentition are quite common. When primary teeth are subjected to trauma, force transmission and/or invasion of the underlying tooth germs lying in close proximity can result in a variety of disturbances in the permanent successors. Few of these disturbances include hypoplasia, dilaceration, or alteration in the eruption sequence and pattern. Dilaceration is defined as an angulation or sharp bend or curve in the linear relationship of the crown of a tooth to its root. A rare case of maxillary left central incisor having crown dilaceration and Vertucci's type II canal configuration with symptomatic periapical periodontitis is reported. Cone beam computed tomography was used for better understanding of the anomaly and complicated root canal morphology. The tooth was successfully managed by nonsurgical root canal therapy and restoration with resin composite to restore esthetics.

## 1. Introduction

Trauma resulting in oral hard and soft tissue injuries is quite common in children especially in the anterior region. The reported prevalence of traumatic injuries in primary teeth is quite high and has shown to range from 11 to 30% [1]. The close anatomic relationship of the permanent tooth germs to the roots of primary teeth makes them highly vulnerable to the impact of trauma. Disturbances in permanent teeth subsequent to trauma are well documented and can range from yellowish brown discoloration to structural alterations like hypoplasia, dilacerations of the crown and/or root, incomplete root formation, crown/root duplication, odontome like malformation, sequestration of tooth germ, and disturbed eruption of the permanent teeth [2, 3]. The type and severity of disturbances are dependent on the direction and amount of force, stage of the developing tooth germs, and their spatial relationship to the roots of primary teeth.

Dilaceration is a rare disturbance in traumatized permanent teeth and constitutes about 3% of the total injuries to the developing teeth [1, 4, 5]. It is defined as an angulation or sharp bend or curve in the linear relationship of the crown of a tooth to its root [6]. The term was first coined by Tomes in 1848 [7]. It usually occurs subsequent to intrusion, avulsion, and subluxation injuries of primary teeth and is seen to commonly affect permanent maxillary central incisors followed by mandibular central and lateral incisors [8, 9]. Crown dilaceration with palatal deviation is more common in maxillary incisors, whereas labial deviation is more common in mandibular incisors [10].

For determining the normal as well as abnormal external and internal morphology of a tooth, intraoral periapical radiographs are an essential diagnostic tool in endodontics. However, a radiograph is a 2-dimensional view of a 3-dimensional structure, and hence, the ability to clearly view complex tooth morphology especially in the buccolingual plane is an inherent limitation of the radiograph. To overcome these limitations, cone beam computed tomography (CBCT) is popularly being used nowadays in endodontics to aid in preoperative diagnosis, optimal treatment planning, and posttreatment assessment. It provides a great deal of information on unexpected and complex anatomy, degree of calcification, direction, number and curvature of canals, aberrant root canal configurations, root resorption, root fractures, perforations, and periapical pathology. Use of CBCT greatly benefits over multiple radiographs by providing detailed information on three dimensional images thereby enhancing accurate assessment while reducing radiation exposure in complex endodontic cases [11].

Maxillary central incisors are teeth mostly known to have a single root and a single canal. The presence of additional canals in these teeth is extremely rare with a reported incidence of only 0.6% [12]. Most reported cases of central incisors with extra canals have shown an association with developmental alterations like macrodontia, fusion, germination, dens in dente, and supernumerary roots [13]. No case of maxillary central incisor having dilacerated crown and two canals has ever been reported.

The purpose of this paper is to present and describe the endodontic management of a maxillary central incisor having crown dilaceration and Vertucci type II canal configuration, diagnosed using radiographic and cone beam computed tomography examination.

## 2. Case Report

A 17-year-old boy reported to the department of Conservative Dentistry and Endodontics with the complaint of pain and discoloration in the maxillary anterior region. The patient's medical history was noncontributory. Dental history indicated trauma to the anterior maxilla at 2-3 years of age due to fall from stairs, following which he had lost his left primary incisors. No dental treatment was undertaken for the same until the patient visited the dental clinic with the complaint of pain in relation to left upper front tooth. His parents confirmed that the permanent successors had erupted with alterations in color and shape and though they were dissatisfied with the esthetics, they had never visited a dentist earlier.

**Figure 1:**
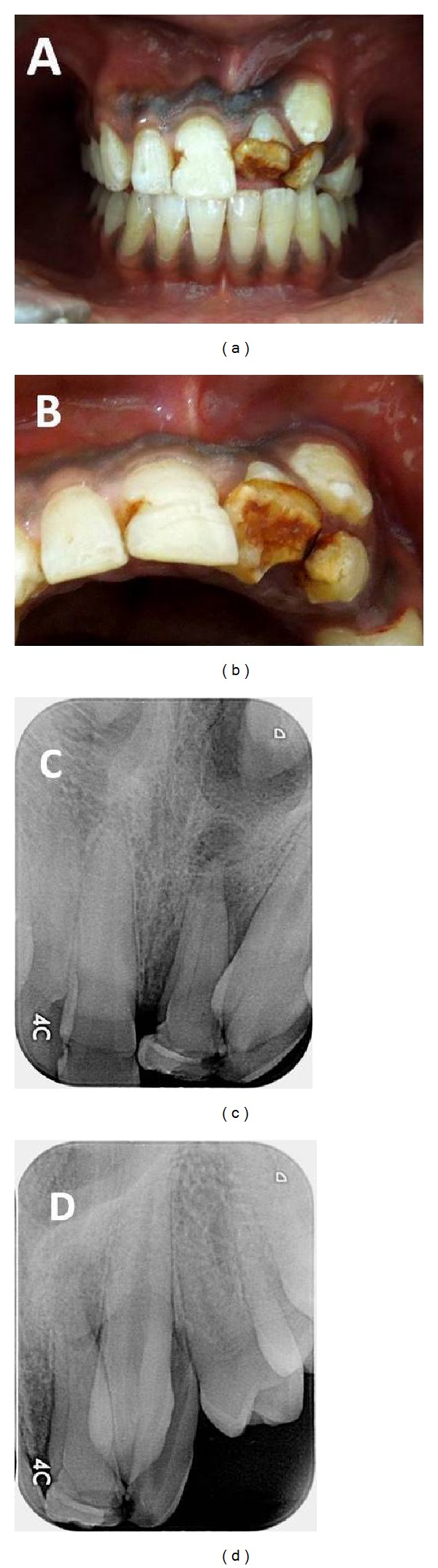
(a) Facial clinical view showing hypoplastic incisors, shortened lateral incisor crown, and mesioangularly placed canine. (b) Incisal clinical view of maxillary incisors showing acute palatal displacement of the crown in left central incisor and deficient enamel on the mesiolingual aspect of the left lateral incisor. (c) Preoperative intraoral periapical radiograph of left maxillary anterior region with anteroposterior projection. (d) Preoperative intraoral periapical radiograph of left maxillary anterior region with angulated projection.

On clinical examination, it was seen that the maxillary right and left central incisors, left lateral incisor, and left canine were the affected teeth. Maxillary left central incisor appeared to have a short clinical crown length with alteration in the incisal and middle thirds of the crown. The affected part of the crown was distinctly hypoplastic with yellowish brown discoloration (Figures 1(a) and 1(b)). On closer examination, it appeared that the coronal part of the crown was bent palatally. When probed, it was possible to penetrate the explorer underneath the crown on the palatal side indicating a hooked like appearance. The crown of left maxillary lateral incisor was rudimentary in size, hypoplastic with yellowish brown discoloration, and deficient on the mesiolingual aspect (Figures 1(a) and 1(b)). Enamel was present only on facial and distal surfaces of the crown. The left maxillary canine displayed a normal appearance but was partially erupted and positioned labial to the crowns of teeth number 9 and number 10 (Figures 1(a) and 1(b)). Slight hypoplasia and discoloration at the junction of the cervical and middle thirds were also evident on the maxillary right central incisor (Figures 1(a) and 1(b)). The right lateral incisor and canine appeared normal in structure. Vitality testing with thermal and electrical pulp tests showed no response in teeth number 9 and number 10. Tenderness on percussion and pain on palpation were noted in relation to tooth number 9.

Intraoral radiographic examination revealed the presence of a radiopaque line in the cervical area of tooth number 9 and also revealed a periapical radiolucency surrounding its root apex. Additionally, tooth number 9 appeared to have two root outlines which led us to suspect the presence of two canals (Figures 1(c) and 1(d)). Angulated radiographs were taken but the exact anatomy of the tooth could not be clearly identified especially because of the interference from the overlapping canine. Tooth number 10 showed incomplete maturation of the tooth with a blunderbuss canal. Its crown and root appeared foreshortened with a slight mesial inclination. Tooth number 11 was well formed but mesioangularly impacted (Figures 1(c) and 1(d)). A circular radiolucent line was seen at the junction of the cervical and middle thirds of tooth number 8 (Figure 1(c)). Coronal to this radiolucent line, the crown appeared to have no abnormality.

**Figure 2:**
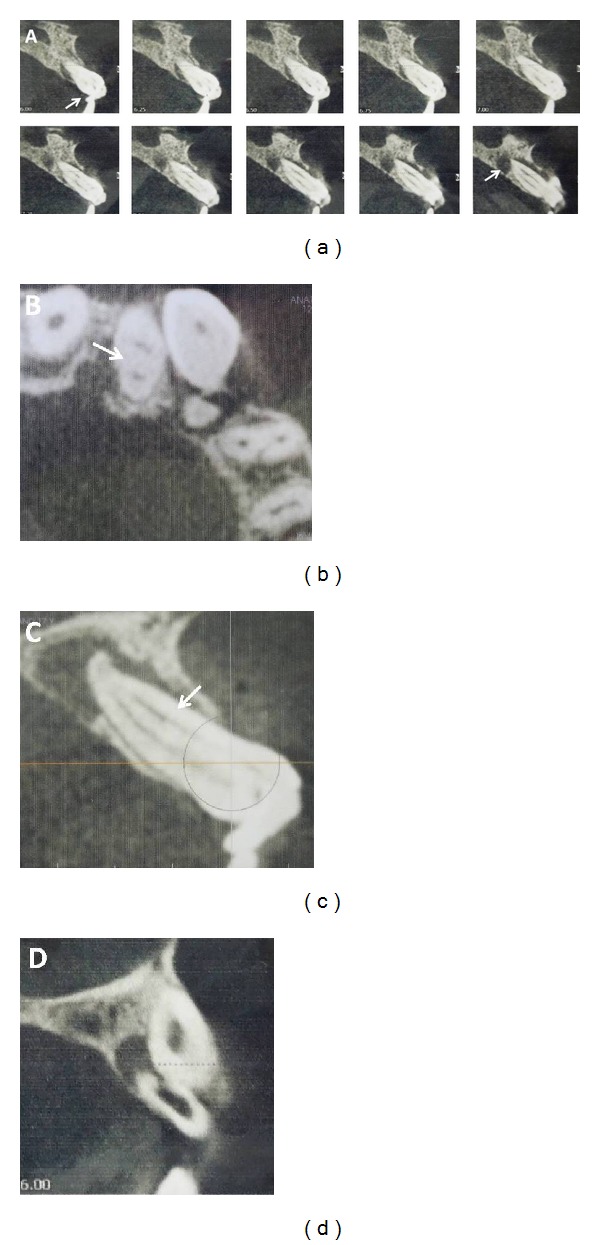
Cross sectional CBCT images showing (a) the presence of a structure which is composed of enamel and dentin in relation to the crown of maxillary left central incisor and also a well-defined periapical radiolucency with respect to its root. (b) Axial image showing two root canals in left maxillary central incisor (c) single root with two distinct root canals, one each on the labial and palatal aspect. (d) Periapical radiolucency and perforation defect in relation to left lateral incisor.

The exact tooth anatomy of tooth number 9 could not be verified on radiographic examination, and hence, to ascertain the variations in tooth anatomy and its root canal system, dental imaging with CBCT (Next Generation i-CAT, Imaging Sciences International, Hatfield, PA, USA) for the affected region was undertaken using a limited field of view with exposure parameters of 120 kV, 5.0 mA, and 0.25 mm voxel size. The images were reconstructed at 0.25 mm thickness increments. Cross sectional CBCT images confirmed the presence of a well-defined periapical radiolucency and the presence of a structure which was composed of enamel and dentin in relation to the crown of tooth number 9 (Figure 2(a)). The tooth had a single root rather than two roots as suspected earlier but had an abnormally greater buccolingual width with two distinct root canals, one each on the labial and palatal aspect (Figure 2(b)). Root dilaceration was also noted at the apex (Figure 2(c)). Additionally, well-defined periapical radiolucency with perforation like defect was also revealed on the labial aspect at the level of the apical third in the root of tooth number 10 (Figure 2(d)). Enlarged pulp chamber and root canal were confirmed on the CBCT images.

Based on these clinical and radiographic findings, a diagnosis of crown dilaceration of left central incisor having symptomatic periapical periodontitis was made. Lateral incisor was seen to be affected with Type IV hypoplasia and asymptomatic periapical periodontitis. It was decided to perform root canal treatment on tooth number 9 and extract tooth number 10 followed by interdisciplinary management for repositioning and esthetic correction of the affected teeth.

## 3. Treatment

**Figure 3:**
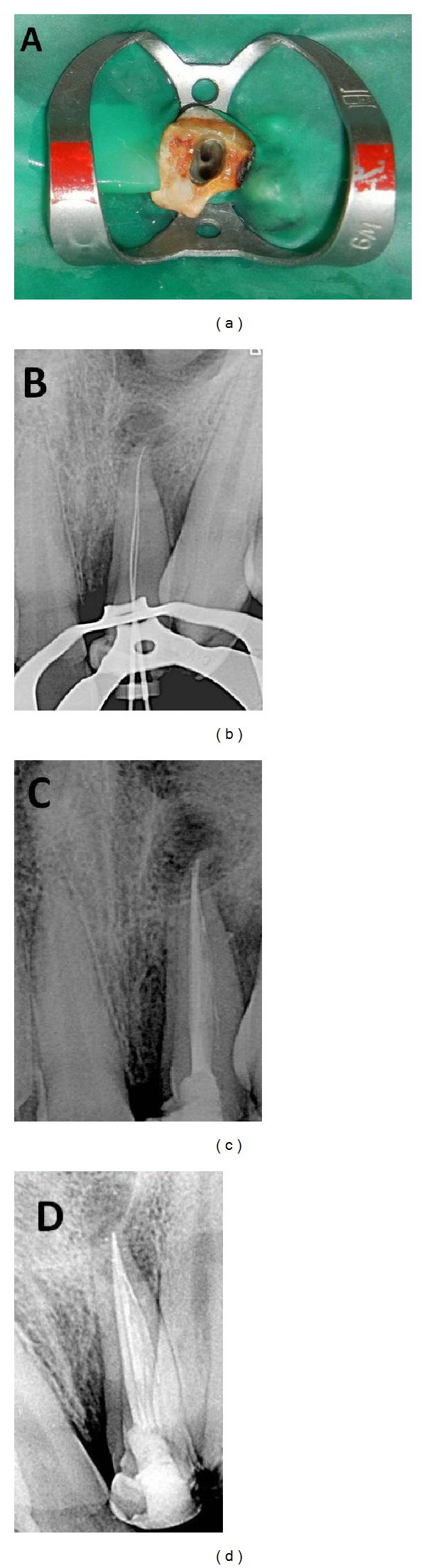
(a) Clinical picture showing two distinct canal openings: one labial and one palatal in the central incisor. (b) Radiographic determination of working length. (c) Postoperative periapical radiograph demonstrating completed nonsurgical root canal therapy. (d) Postoperative periapical radiograph with angulated projection.

After complete explanation of the treatment procedure, risks, and prognosis, an informed consent was obtained. Impressions and study models were made and nonsurgical root canal therapy was initiated in tooth number 9. Access cavity was prepared using Endo Access Kit (Dentsply) under rubber dam isolation, and both buccal and palatal canals were located under magnification using dental loupes (2.5x, Daray, Derbyshire). The pulp chamber when opened was found to be greatly diminished in size either due to calcification or due to the developmental anomaly. The root canals were also highly calcified with extremely narrow openings posing great difficulty in locating them. The canals were initially instrumented with K flex number 6, 8, and 10 files using 17% EDTA (Figure 3(a)). Working length of 22.5 mm was determined using electronic apex locator (Root ZX, J morita Mfg Corp., Japan) and confirmed radiographically (Figure 3(b)). The root canals were cleaned and shaped with size 15–40 Ni-Ti K files (Dentsply Maillefer, Switzerland) using step back technique. The canals were copiously irrigated with 2.5% sodium hypochlorite solution. After the canals were properly dried with paper points, calcium hydroxide (Metapex, Meta Biomed Co., Ltd., Korea) was placed inside the canals and the access cavity was temporarily sealed with IRM (Caulk, Dentsply, USA). After two more similar visits of the patient spaced at 1-week intervals, when the patient was completely asymptomatic, the canals were rinsed with saline and dried using absorbent paper points. Canals were obturated with gutta-percha (Dentsply Maillefer, Switzerland) using cold lateral compaction technique and AH Plus resin as a sealer (Dentsply, De Trey, Germany) (Figure 3(c)). The access cavity was permanently restored with resin composite.

**Figure 4:**
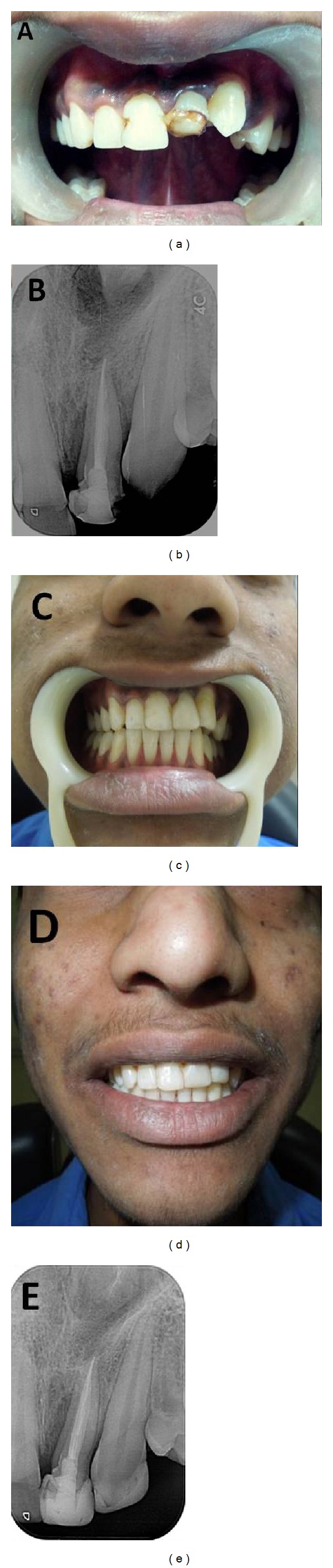
(a and b) Clinical and radiographic examination at 12 months revealed spontaneous eruption of canine and its more vertical placement. (c and d) Restoration of central incisor and canine with resin composite: a clinical view. (e) Restoration of central incisor and canine: a radiographic view.

Tooth number 10 was extracted under local anesthesia. The patient was recalled after 3 months for evaluation of periapical healing with respect to tooth number 9. At 3 months, resolution of periapical radiolucency was seen in tooth number 9, and hence the patient was referred to the department of Orthodontics for orthodontic correction of teeth. The patient however failed to report on his scheduled appointment. On contacting him, it was learnt that he had left for his native place without having started any further treatment. He reported again to the department after 8 months. It was observed on clinical examination that spontaneous eruption of tooth number 11 had taken place and was now more vertically placed and only a little short of the occlusal plane (Figures 4(a) and 4(b)). Orthodontic treatment was now not deemed necessary. It should be noted here that the canine showed delayed eruption at the age of 17.5 years, whereas its normal eruption time is around 13-14 years. Esthetic correction was performed in both teeth number 9 and number 11 using restorative treatment (Figures 4(c), 4(d), and 4(e)). Teeth number 8 and number 9 were esthetically corrected with composite using incremental technique. Tooth number 11 was shaped into a lateral incisor using resin composite. Restored teeth were finished and polished. The patient was referred to the department of Prosthodontics for restoration of the edentulous space mesial to first premolar.

## 4. Discussion

Traumatic injuries during the primary dentition stage are quite common. Primary tooth trauma resulting in developmental disturbances in permanent successors has shown to have a prevalence that ranges from 12% to 74% [1, 2, 14]. The direction and magnitude of impact, age of the patient at the time of injury, stage of the developing tooth germs, and their anatomic proximity to the roots of primary teeth are critical factors in determining the effect of injury and its manifestations in permanent teeth [15]. Do Espírito Santo Ja'como and Campos (2009) have reported enamel discoloration/hypoplasia (46.08%) and eruption disturbances (17.97%) as the most common developmental disturbances in permanent teeth [16]. The same group has also reported 9% incidence of crown dilaceration, 3 times higher than the results presented by other studies [1, 5].

Dilaceration can occur anywhere along the length of the tooth, that is, the crown, cementoenamel junction, length of the root, or root apex. Crown dilaceration has usually shown to have a greater occurrence following intrusion or avulsion of primary teeth, and the most affected age group seen is between 1.5 and 3.5 years at the time of injury. Normally, at the age of 2-3 years, the tooth germs of permanent maxillary incisors are located above and palatal to the root apices of primary teeth. When a force is applied to the labial surface of a primary maxillary central incisor crown, the root moves labially, with less chance of disturbing the permanent tooth germ, whereas when a force is applied to the palatal surface of the primary maxillary incisor crown as in the case of traumatic avulsion, the root moves palatally, thereby causing trauma to the underlying tooth germ [17]. Affected teeth are usually seen to erupt either labially or palatally [8].

Pathology of crown dilaceration can be explained by the theory of displacement of the mineralized portion of the tooth in relation to the dental papilla, inner and outer enamel epithelium, and cervical loops [18]. Facially, the stretched inner enamel epithelium is able to induce differentiation of new odontoblasts; hence, dentin formation is not affected but enamel formation is usually affected. Consequently, a horizontal band of dentin without enamel on the facial aspect is evident. The facial cervical loop either is not injured or regenerates, and normal amelogenesis continues apical to the trauma site. On the lingual aspect, the displaced inner epithelium and ameloblasts form a cone of hard tissue which usually projects into the pulp canal.

In the present clinical case, traumatic force resulting from the avulsion injury at 2-3 years seemed to affect the left central and lateral incisors the most, with a lighter impact on the right central incisor. The tooth germ of canine was also possibly displaced mesioangularly. The impact appeared to be directed at the incisolabial surface of the left central incisor tooth germ resulting in its palatal displacement. The characteristic hook like appearance of the dilacerated crown was well discernible in the CBCT cross-sectional axial slices. The tooth also erupted in slight labioversion. Additionally, CBCT images revealed a single root in left central incisor with an abnormally greater buccolingual width in the cervical and middle thirds and Type II Vertucci canal configuration. Here, maxillary right central incisor showed a normal Vertucci Type I canal configuration, strongly indicating that localized trauma was the probable etiology for the occurrence of additional canal in the left maxillary central incisor.

Aberrations in root canal anatomy could possibly be attributed to the stretching apart of the facial and lingual cervical loops resulting in the involution of the hertwigs epithelial root sheath on the proximal sides. This could also possibly explain the greater buccolingual width of the root with deep developmental grooves on the proximal surface which was visible on the radiograph as two root outlines. The presence of dilacerated crown with hypoplastic enamel and dentin would have predisposed to pulpal and periapical involvement as it served as a nidus area for microorganisms and their easier penetration into the open dentinal tubules.

In the current case, traumatic injury also affected the left lateral incisor tooth germ resulting in Type IV hypoplasia (enamel discoloration, abnormal coalescence, some parts of enamel missing) and arrested development of the crown [19, 20]. The tooth probably turned nonvital soon after eruption because of the defective enamel and open dentinal tubules which promoted bacterial entry into the pulp space. Because of this early involvement of the pulp, dentin formation ceased and root growth was arrested. The resultant immature root had a blunderbuss canal.

Circular enamel hypoplasia is a disturbance of the enamel resulting in a line defect surrounding the crown of the injured tooth and most frequently occurs as a result of trauma in children around the age of two years [21, 22]. Since only the cervical part of the right central incisor crown would have formed by the age of 2-3 years, defective and hypoplastic enamel seen at the junction of cervical and middle thirds of the crown clearly indicated that the impact of injury had also affected this tooth, though to a lesser extent.

Radiographic examination is an integral part of diagnosis, treatment planning, and management in endodontics. Angulated radiographs and digital radiography compared to single radiographs greatly improve the ability to identify variations in tooth anatomy [23]; yet the information provided is limited because of the superimposition and distortion of structures and the fact that it is a 2-dimensional image [24]. Use of CBCT can help overcome these limitations associated with intraoral and panoramic radiography. CBCT can be used to highlight specific anatomic regions for diverse diagnostic tasks by reconstructing the projection data to provide interrelational images in 3 planes. The elimination of anatomic noise facilitates the assessment of a number of features important in endodontic diagnosis and treatment [25].

In this specific case, the presence of crown dilaceration, additional root canal, and diminished pulp chamber size in left central incisor were confirmed on CBCT. It was essential to avoid perforating the root while locating and negotiating the canals. Endodontic success was achieved despite the difficult internal anatomy of the canal system emphasizing the need for correct diagnosis and determination of morphological variations before treatment onset. The presence of a periapical pathology with a labial perforation defect in relation to the root of the left lateral incisor was additionally detected on the CBCT images only. This information was otherwise missed on the conventional radiographs because of the overlapping mesioangularly placed canine.

Disturbances in the permanent dentition subsequent to trauma of primary teeth often dictate an interdisciplinary management depending on severity and extent. The case presented here showed multiple teeth affected by crown dilaceration, hypoplasia, and improper eruption. Orthodontic repositioning for correction was not considered necessary. It was possible to treat the case by endodontic treatment followed by alignment and leveling with esthetic restorative treatment.

## 5. Conclusion

Cone beam computed tomography has greatly facilitated safe and effective endodontic treatment by serving as an important tool for assessment of complex internal and external anatomy of the tooth. By establishing a correct diagnosis and adhering to the basic principles of endodontic treatment, it is possible to achieve endodontic success in teeth with variations in tooth anatomy.
